# Evaluation of Cystoid Change Phenotypes in Ocular Toxoplasmosis Using Optical Coherence Tomography

**DOI:** 10.1371/journal.pone.0086626

**Published:** 2014-02-05

**Authors:** Yanling Ouyang, Uwe Pleyer, Qing Shao, Pearse A. Keane, Nicole Stübiger, Antonia M. Joussen, Srinivas R. Sadda, Florian M. Heussen

**Affiliations:** 1 Charité, University Medicine Berlin, Department of Ophthalmology, Berlin, Germany; 2 NIHR Biomedical Research Centre for Ophthalmology, Moorfields Eye Hospital NHS Foundation Trust and UCL Institute of Ophthalmology, London, United Kingdom; 3 Doheny Eye Institute and Department of Ophthalmology, Keck School of Medicine of the University of Southern California, Los Angeles, California, United States of America; Medical University Graz, Austria

## Abstract

**Purpose:**

To present unique cystoid changes occurring in patients with ocular toxoplasmosis observed in spectral domain optical coherence tomography (OCT).

**Methods:**

Forty-six patients (80 eyes) with a diagnosis of ocular toxoplasmosis, who underwent volume OCT examination between January 2005 and October 2012, were retrospectively collected. Review of clinical examination findings, fundus photographs, fluorescein angiograms (FA) and OCT image sets obtained at initial visits and follow-up. Qualitative and quantitative analyses of cystoid space phenotypes visualized using OCT.

**Results:**

Of the 80 eyes included, 17 eyes (15 patients) demonstrated cystoid changes in the macula on OCT. Six eyes (7.5%) had cystoid macular edema (CME), 2 eyes (2.5%) had huge outer retinal cystoid space (HORC), 12 eyes (15%) had cystoid degeneration and additional 3 eyes (3.75%) had outer retinal tubulation due to age related macular degeneration. In one eye with HORC, the lesion was seen in the photoreceptor outer segment, accompanied by photoreceptor elongation and splitting. Three eyes presented with paravascular cystoid degeneration in the inner retina without other macular OCT abnormality.

**Conclusions:**

In this study, different phenotypes of cystoid spaces seen in eyes with ocular toxoplasmosis using spectral domain OCT (SD-OCT) were demonstrated. CME presented as an uncommon feature, consistently with previous findings. Identification of rare morphological cystoid features (HORC with/without photoreceptor enlongation or splitting) on clinical examination had provided evidence to previous experimental models, which may also expand the clinical spectrum of the disease. Cystoid degeneration in the inner retina next to the retinal vessels in otherwise “normal” looking macula was observed, which may suggest more often clinical evaluation for those patients. Further studies are needed to verify the relevance of cystoid features seen on SD-OCT in assisting with the diagnosis and management of ocular toxoplasmosis.

## Introduction

Ocular toxoplasmosis is the most common form of posterior uveitis in otherwise healthy individuals, leading to legal blindness in at least one eye in approximately 25% of cases [1]. It occurs mainly in children and young adults, with significant morbidity, and thus has considerable socio-economic implications. In addition, more severe disease commonly develops in immunosuppressed patient populations, particularly those with the acquired immune deficiency syndrome. In such patients, the occurrence of ocular toxoplasmosis may be an important indicator of intracranial involvement [2].

The pathogenesis of ocular toxoplasmosis – in particular pertaining to mechanisms of disease recurrence – is poorly understood [3]. In humans, the majority of recurrent retinal lesions arise at the borders of old retinochoroidal scars. The appearance of such lesions may be the result of parasite release from tissue cysts, with invasion and destruction of adjacent cells [3], [4]. The immune response induced by these parasites may also play a significant role [3]. Advances in *in vivo* imaging of retinal lesion morphology, especially optical coherence tomography (OCT), combined with known histopathology, may thus enable further understanding of disease progression mechanisms.

The introduction of spectral domain OCT (SD-OCT) has significantly improved the speed and sensitivity of OCT devices. These improvements allow excellent visualization of the neurosensory retina and have facilitated identification of numerous, hitherto unknown, retinal disease features [5]. In this study, we analyse the phenotype of cystoid changes seen on macular OCT, in an effort to gain a better understanding of the evolution of these lesions and the pathogenesis of ocular toxoplasmosis.

## Materials and Methods

### Baseline Data Collection

Data were retrospectively collected from consecutive patients attending the Department of Ophthalmology at Charité, University Berlin, between January 2005 and October 2012, with a diagnosis of ocular toxoplasmosis. For inclusion in the study, all patients were required to have undergone volume OCT scanning with spectral domain OCT (Spectralis, Heidelberg Engineering, Germany). Diagnoses were based on clinical characteristics consistent with toxoplasma retinochoroiditis (foci of retinal necrosis with associated retinal inflammation), in the absence of other identifiable causes. In addition, confirmatory laboratory testing included serology and/or aqueous humor analysis (detection of Toxoplasma antibodies or detection of T. gondii DNA using polymerase chain reaction). Patients with AIDS or other immune system diseases, or receiving immunosuppressive drugs (other than corticosteroids specifically for ocular toxoplasmosis), were excluded [6]. Information regarding age, sex, ethnicity, history of ophthalmic diseases or surgeries, ophthalmic diagnosis, and lens status were also collected. Best-corrected visual acuity (VA) was measured using Snellen VA charts. Approval for data collection and analysis was obtained from the institutional review board of the Göttingen ethics committee. The research adhered to the tenets set forth in the Declaration of Helsinki. Written consent was given by the patients for their information to be stored in the hospital database and used for research.

### OCT Scanning Protocols and Analysis

In each case, macular OCT scans were obtained (i.e., image sets centered approximately on the fovea) using Spectralis OCT. Raw OCT data were exported from the imaging system and imported into validated custom grading software (3D-OCTOR, Doheny Image Reading Center (DIRC), Los Angeles, CA) [7] for further review.

### OCT Grading Methodology

**Figure 1:**
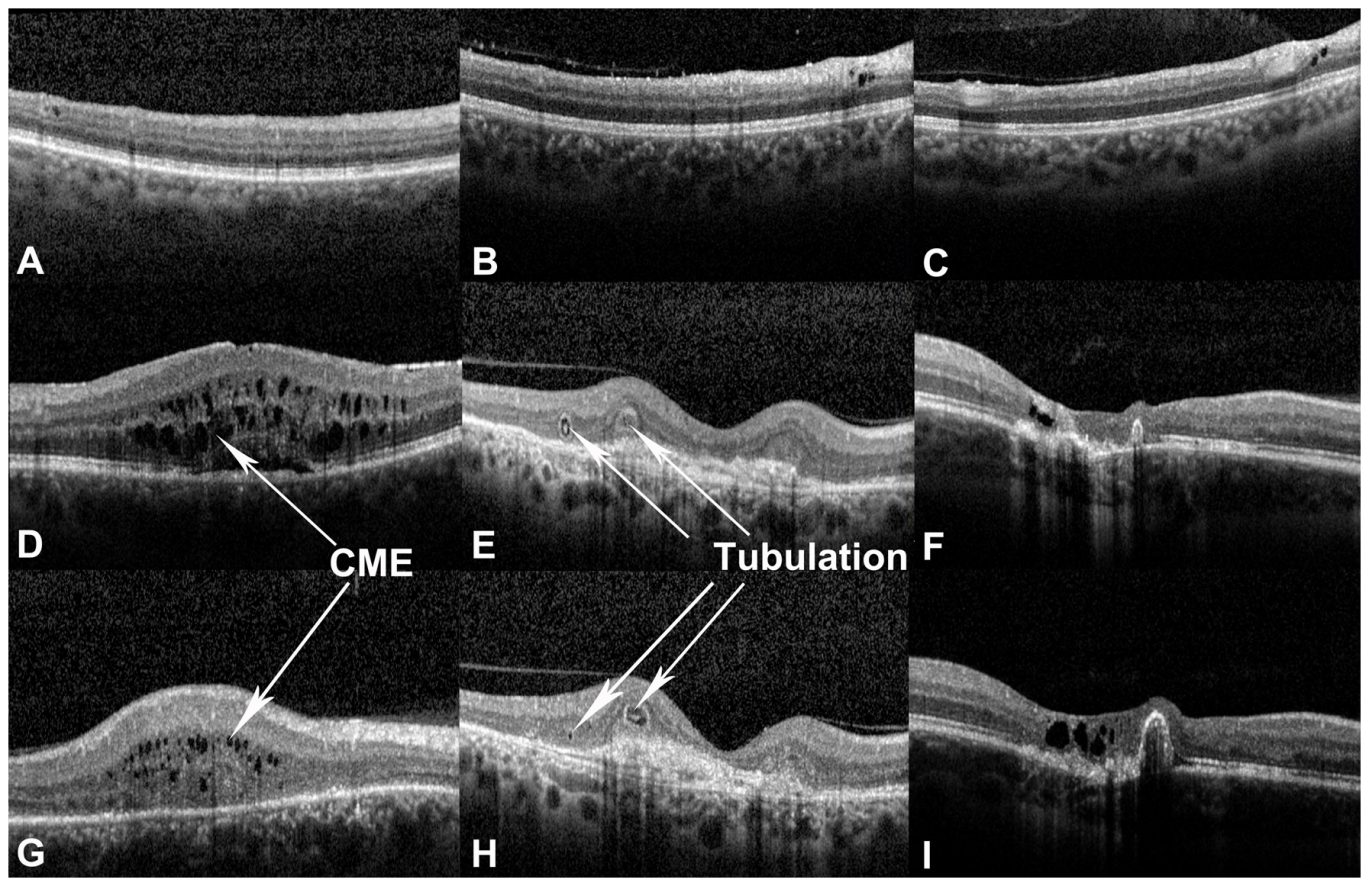
Case examples of cystoid space (CS) phenotypes in ocular toxoplasmosis as seen on optical coherence tomography (OCT). [**A–C**] Examples of cystoid degeneration next to retinal vessels in the inner retina. Figure B was from the right eye of an 18 year-old woman. She was with a previous diagnosis of ocular toxoplasmosis and presented with decreased vision of one week's duration in the same eye. VA in the right eye was 20/25. Examination of the fundus of the right eye revealed vitreous cells, and an active creamy yellow lesion (about 1/3 disc diameters in size) with retinal hemorrhage on the nasal aspect of the optic disc with temporal optic nerve head swelling. Macular was with no obvious abnormality. OCT imaging of the right macula demonstrated an ERM with a few punctate spots in the vitreous, intraretinal CSs (in one B-scan, adjacent to a retinal vessel), along with absence of other retinal abnormalities. [**D, G**] Examples of cystoid macular edema (CME). Figure G was from the right eye of a 51 year-old man presenting for a regular follow-up examination of ocular toxoplasmosis. He had previously been diagnosed with ocular toxoplasmosis affecting the right eye at 17 years old. In his right eye, VA was 20/125 and anterior segment examination was unremarkable. Dilated fundus examination revealed a central macular scar with no findings suggestive of disease activity. OCT imaging showed multiple small CSs in the OPL and ONL, with increased retinal thickness, and categorized as CME. [**E, H**] Examples of outer retina tubulation. Figure E and H were from the right eye of a 70 year-old man, with a previous diagnosis of bilateral ocular toxoplasmosis. On examination, his VA was 20/20 on the right eye. Fundus examination revealed an old macular scar in the right eye without signs of disease activity. The patient had been diagnosed with choroidal neovascularization (CNV) secondary to age related macular degeneration in 2011 and treated with three injections of intravitreal ranibizumab (Lucentis, Genentech, USA) to the right eye. OCT imaging of the right eye demonstrated outer retina tubulation in the degenerated outer retina. [**F,I**] Examples of cystoid degeneration next to old scars.

**Figure 2:**
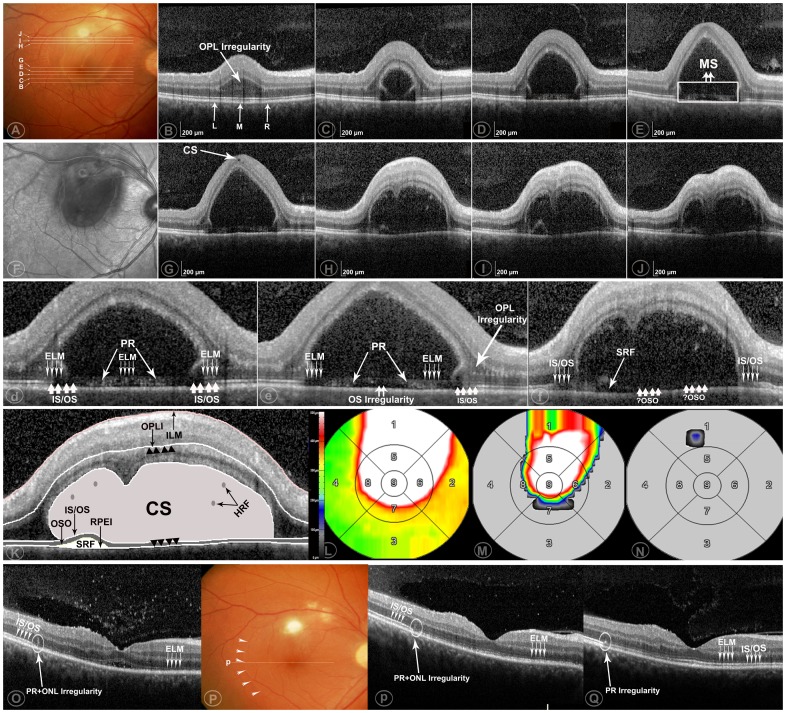
Case example of huge outer retinal cystoid space (CS) as seen on optical coherence tomography (OCT) in a patient with ocular toxoplasmosis. Two days after initial presentation [**A–N**] and at subsequent follow-ups [**P–Q**] (note: [**d–f**] is the magnified view of [**D–F**]). The OCT scans were taken within a 20°×15° (5.8×4.4 mm) area. Distance between adjacent B-scans (B-scans B, C, D, E, G are adjacent; H, I, J are adjacent) was 243 µm. Two days after initial presentation, a membranous structure is seen on OCT. One part of this structure is seen lying over the retinal pigment epithelium (RPE) and forming the floor of the huge outer retinal CS (HORC) [**B–E, G**], while another part is separated from the RPE by an accumulation of subretinal fluid (SRF) [**H–J**]. The line representing the external limiting membrane (ELM) is distinct in the area unaffected by HORC; however, it is continuous with the upper border of the highly reflective membranous structure in the area affected by HORC [**D–F, d–f**]. The photoreceptor inner segment/outer segment junction (IS/OS junction) is distinct in the area unaffected by HORC, becoming less distinct in the area with overlying HORC. Photoreceptor IS/OS disruption and outer segment (OS) irregularity is also observed [**E, G–J, e–f**]. The retinal thickness map [**L**], mean HORC height map [**M**], and the mean maximum SRF height map [**N**], are shown for the Early Treatment Diabetic Retinopathy Study (ETDRS) grid. The presumed interpretation of the structural changes are also shown (the outer retina is the area between the black arrows) [**K**]. OCT images obtained 15 days later demonstrate that the HORC is no longer present [**O**]. Punctate hyperreflective foci are seen in the posterior vitreous overlying this area. The ELM is almost completely distinct in all B-scans. In contrast, the photoreceptor IS/OS junction is still not fully distinct. Irregularity of the PR and outer nuclear layer (ONL) presenting as local hyperreflective foci is seen at the previous junction of the HORCs with the normal retina. This feature persists at subsequent visits [**P–Q**]. A hyporeflective space between the ELM or presumed IS/OS and inner boundary of the RPE is observed subfoveally, with possible hyperreflective material above RPE. Subsequent OCT images were obtained approximately one month [**P**] and two months [**Q**] following initial presentation. No HORC or SRF is seen at these points. The photoreceptor IS/OS junction is more clearly seen, with a thickness value of 68 µm [**Q**].

**Figure 3:**
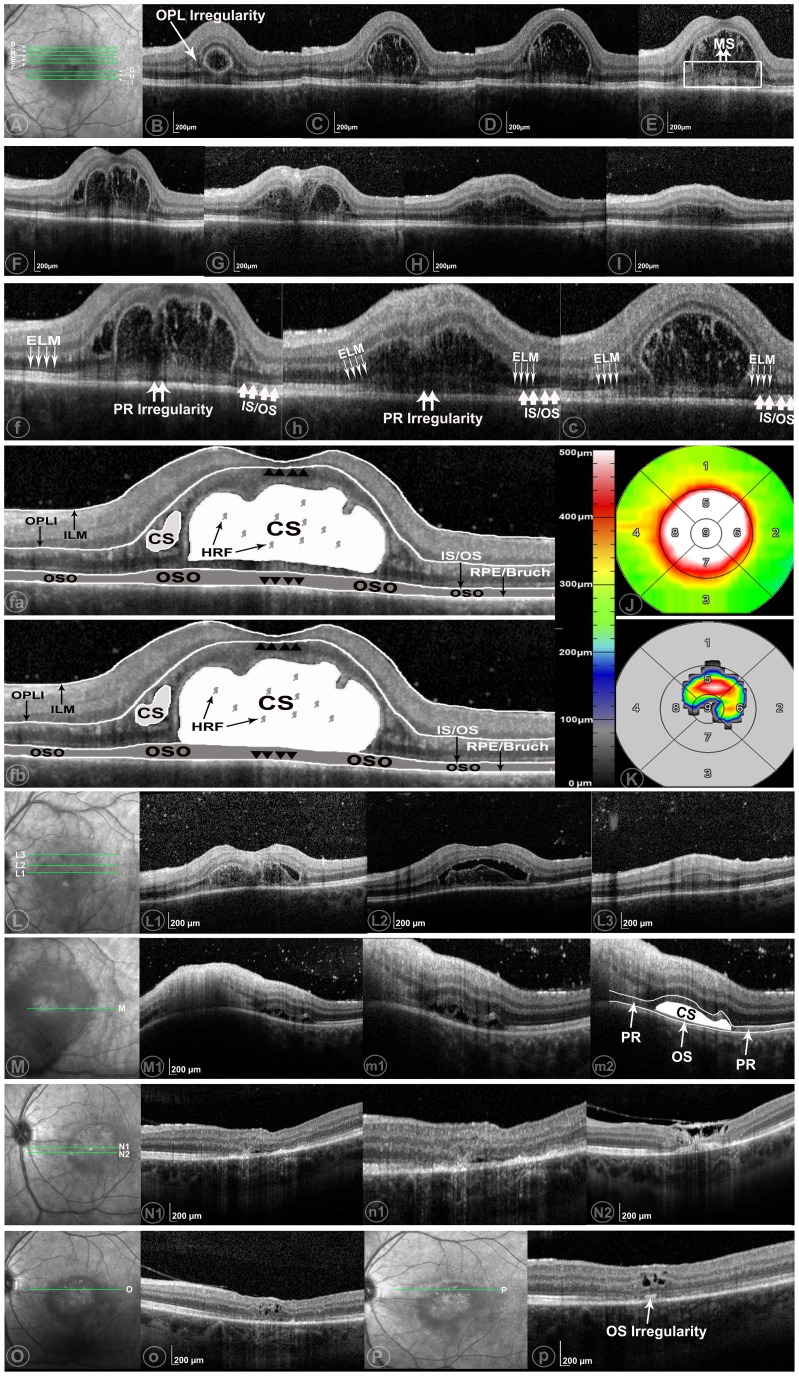
Case example of huge outer retinal cystoid space (CS) as seen on optical coherence tomography (OCT) in a patient with ocular toxoplasmosis. **Part 1** (Part A–K) was present with huge outer retinal CS (HORC) on June 29 2012 (f, h, c were the magnified views of F, H, C). The OCT scans were taken within a 20°×15° (5.9×4.4 mm) area. Distance between adjacent B-scans (B-scans B, C, D, E, F are adjacent; G, H, I are adjacent) was 244 µm. Predominant hyper reflective foci (HRF) were seen within the HORCs. One part of a membranous structure lies on the retinal pigment epithelium (RPE) (C–I) and possibly as a lower border of HORCs (C–D). The line representing the external limiting membrane (ELM) is only distinct in area without HORCs (B–I). Photoreceptor inner segment/outer segment junction (IS/OS) elongation and splitting were seen in F, H. IS/OS disruption and outer segment (OS) irregularity were observed (F, H). The retinal thickness map (J) and the mean maximum height of HORCs (K) were shown in on the Early Treatment Diabetic Retinopathy Study (ETDRS) grid. The presumed interpretations of the structural changes were shown in fa (CS in the outer retina, excluding photoreceptor inner segment) and fb (CS in the outer retina, involving photoreceptor inner segment)(outer retina is the area between the black arrows). **Part 2**: Part L-L3 were the examples of OCT images 4 days later. HORCs became separated from the above retina, forming a hyporeflective empty space (L1-2). The reflectivity inside the HORCs became more hyperreflective (L1). OPL irregularity with increased thickness and change of reflectance was also seen (L3). Part M-m2 were OCT images on July 19 2012 (m1 and m2 are the magnified views for M1). Inner and outer retina necrosis happened at the location where previous HORCs existed (M1). A new HORCs presenting between IS/OS and RPE was seen. A membranous structure locating under this HORCs but above RPE with unique thickness was labeled as OS (m2). Part N-N2 were the examples on August 30 (n1 was the magnified view of N1). HORCs presented in M became smaller (N1), but clearly between IS/OS and RPE inner boundary. Retina degeneration, especially in the subfoveal area, along with thickened and detached posterior hyaloid was shown in N2. Part O and Part P were the OCT images on October 4 2012 and October 29 2012, respectively. Cystoid degeneration was seen in both visits. A local hypereflective band between IS/OS and RPE inner boundary (p) were present at the location with HORCs seen in M and N, which implied previous disruption of OS.

Two graders (Y.O., F.M.H.), certified for the assessment of OCT images at the DIRC, evaluated each OCT image set independently. The presence or absence of cystoid changes was firstly assessed [8]. When cystoid changes were identified, their axial location was then documented according to a pre-existing OCT grading methodology [9]. The phenotypes of cystoid lesions were also assessed as 1) cystoid macular edema (CME), 2) outer retinal cystoid degeneration due to atrophy (i.e. outer retinal tubulation), [5] 3) cystoid degeneration due to other causes (Figure 1), 4) huge outer retinal cystoid space (CS) (Figure 2,3).

A reading center open adjudication process was applied to obtain the final results from the dual grading [10].

### Quantitative Analysis of OCT Images:

For OCT sets containing huge outer retinal CS (HORC), manual segmentation was performed, which allowed both quantification of retinal thickness and multiple measurements of HORC and subretinal fluid (SRF) (including maximum and minimum height, area and volume). Quantitative parameters were then reported based on the Early Treatment Diabetic Retinopathy Study (ETDRS) grid or the entirety of the scanned area.

In Patient 1 (Figure 2), a membranous structure was present in addition to the HORC. For this case, the thickness of membranous structure, PR thicknesses on the left and PR thicknesses on the right were documented. The thickness of the membranous structure was measured at the center of HORC in each available B-scan (example in Figure 2B as M). PR thickness was defined as the distance between the outer boundary of the external limiting membrane (ELM) and the inner boundary of the RPE. PR thickness on the left/right was measured on the left/right side of the area unaffected by HORC with distinct ELM and closest to the center of HORC (example in Figure 2B as L and R, respectively). In B-scans without HORC, PR thickness in the center of the B-scan was also measured.

### Data Analysis and Statistical Methods

Clinical and imaging data were analyzed with frequency and descriptive statistics. Statistical analysis was performed using commercially available software SPSS (version 19.0, SPSS Inc., Chicago, Illinois, United States). P values<0.05 were considered statistically significant.

## Results

### Baseline Characteristics

One hundred and fifty patients with a diagnosis of ocular toxoplasmosis were seen in our tertiary center within the study period. Altogether, 46 patients (80 eyes; 204 visits) had undergone volume OCT scanning and were included in the study. Of these, all had clinical characteristics consistent with ocular toxoplasmosis. The mean patient age was 39.5±16.3 years (range, 15–78 years). 23 patients (50%) were female, while 23 (50%) were male.

All OCT images included in the study met reading center criteria for sufficient image quality. A total of 137 visits had abnormalities seen on OCT scans, out of which, 112 visits had lesions involving the fovea.

### Range of Cystoid Changes

Out of 80 eyes included in the study, 17 eyes (15 patients, 39 visits) had cystoid changes seen in the macula on volume OCT scanning: 6 (7.5%) with CME, 3 (3.75%) with outer retinal tubulation, 2 (2.5%) with HORC, and 12 (15%) with cystoid degeneration due to other causes (6 eyes with more than one cystoid phenotype present in the same OCT image set).

Three eyes with outer retinal tubulation were due to late degenerative change after treatment of choroidal neovascularization (CNV) secondary to age-related macular degeneration (AMD). One eye presented with HORCs located in the photoreceptor outer segment (OS) accompanied with an outer plexiform layer (OPL) cyst and photoreceptor elongation and splitting. Three eyes presented with cystoid degeneration in the inner retina next to the retinal vessels without other retinal/choroidal abnormality in the macula.

### HORC – Representative Case Studies

#### Patient 1

A 28 year-old Caucasian male, with a previous diagnosis of recurrent ocular toxoplasmosis in the right eye, presented with a new onset scotoma in the same eye. On presentation, VA was 20/20 in each eye. Dilated fundus examination revealed a whitish-yellow inflammatory lesion near an atrophic and pigmented retinochoroidal scar in the right eye, with no obvious abnormality in the left eye. The patient was commenced on oral clindamycin. Two days later, the patient represented with decreased VA (20/200) in the right eye. Fundal examination revealed the presence of new retinal hemorrhage and edema accompanying the inflammatory lesion and involving the fovea (Figure 2A).

On OCT scanning, the inner retina was largely unremarkable with the exception of a small CS seen on a single B-scan. In the outer retina, a huge CS posterior to the inner boundary of the OPL was seen, termed HORC. Only a few spots of hyper reflective foci (HRF) were observed inside the HORC. Irregularity of the photoreceptor OS was also seen. For measurements, HORC was observed in 8 B-scans, with a mean thickness of membranous structure as 67.1±3.7 µm (range: 61–70 µm, n = 8), mean PR thicknesses on the left as 67.0±2.2 µm (range: 62–69 µm, n = 8) and mean PR thicknesses on the right as 66.1±3.3 µm (range: 61–69 µm, n = 8). Statistically, there was no significant difference among PR thicknesses on the left, PR thicknesses on the right and the mean thickness of membranous structure (p = 0.119, nonparametric testing). The mean thickness of the membranous structure was also similar to subfoveal PR thickness in the fellow eye (67 µm).

The patient continued treatment with oral clindamycin. Approximately three weeks following initial presentation, HORC was no longer seen on OCT. Approximately two months following initial presentation, subfoveal PR thickness increased to 68 µm. At the most recent follow-up visit, the patient's VA had recovered to 20/20 (Table 1, Figure 2).

**Table 1:** Clinical Examinations for Case 1.

Date of visits	September 3, 2012 (Fig. 2. A–J)	September 18, 2012 (Fig. 2O)	September 28, 2012 (Fig. 2P)	October 23, 2012 (Fig. 2Q)
**Snellen Visual Acuity**	20/100	20/32	NA	20/25
**Anterior Examination**	within normal limits	Vitreous cell 2++	NA	within normal limits
**Dilated Fundus Examination**	new retinal hemorrhage and edema involving the fovea, accompanied by the typical whitish-yellow inflammatory lesion	absent for hemorrhage or edema; inflammatory lesion similar compared to before	newly present circular line at the border of previous edema; absent for hemorrhage or edema; whitish-yellow lesion without sign of inflammation	no obvious change compared to September 28
**OCT Features** Vitreous	a few punctate spots	prominent punctate spots	less punctate spots	no punctate spots
IR	one small hyporeflective space	within normal limits	within normal limits	within normal limits
OPL	increased thickness with hyporeflectance	within normal limits	within normal limits	within normal limits
HORC	present with a few dots of HRF	absent	absent	absent
ELM	mostly intact but invisible in H-J	intact	intact	intact
IS/OS	not clearly visible in H-J	partially intact foveal disruption	intact subfoveally but thinner	Intact subfoveally, normal thickness
OS	irregularity (E,G)	irregularity	mostly intact	mostly intact
SRF	present (H-J)	absent or questionable present	absent	absent
**OCT measurements** Mean ±SD (range), µm Mean retinal thickness (central subfield)	887.7±86.0 (695.4–1021.6)	208.1±44.5 (117.0–304.4)	215.6±28.3 (156.1–268.3)	228.3±29.2 (163.8–354.8)
Mean HORC height (central subfield)	603.2±114.6 (305.2–755.7)	0	0	0
Maximum SRF height	14.0±31.1 (0–154.2)	0 or 4.8±10.5 (0–58.5)	0	0

IR = intra retina; OPL = outer plexiform layer; HORC = huge outer retinal cystoid space; ELM = external limiting membrane; PR = photoreceptor layer; IS/OS = PR inner segment-outer segment junction; OS = PR outer segment; SRF = subretinal fluid; NA = not available; OCT = optical coherence tomography.

#### Patient 2

A 23 year-old Caucasian woman, with previous history of recurrent ocular toxoplasmosis affecting the left eye, presented with decreased vision in the same eye. On presentation, VA was 20/15 in the right eye and 20/25 in the left eye. Fundus examination of the left eye showed pigmentary changes in the macula unchanged from previous visits, but was otherwise unremarkable. Right fundus examination was normal. OCT imaging of the left eye revealed only the presence of an ERM. Subfoveal PR thickness was 83 µm.

On examination approximately two months later, the patient had a VA of 20/160 in the left eye. On OCT, ERM and punctate hyperreflective spots were seen in the posterior vitreous. The OPL was thickened with focal areas of hyporeflectivity consistent with CSs. Again, a HORC was seen. Inside the HORC, many discrete HRF of irregular shape were observed. The mean retinal thickness in the foveal central subfield was 533.2±26.9 (range, 475.9–600.6) µm. The mean height of the HORC in the foveal central subfield was 152.5±95.2 (0.09–385.0) µm (Figure 3).

On examination four days later, necrosis of the outer retina, consisting of a hyporeflective empty space, was seen on OCT. The mean retinal thickness in the foveal central subfield was 480.7±32.7 (range, 405.8–526.4) µm. The mean height of the HORC was 249.8±45.0 (range, 70.2–307.4) µm. On examination two weeks later, the previously visible HORC had disappeared completely; however, a new hyporeflective space appeared within PR, more precisely, within OS. Necrosis in the inner and outer retina in the fovea was seen, with total retinal destruction in the fovea at subsequent visits. The patient's final VA was 20/125 (Figure 3).

## Discussion

In this study, the presence and phenotypes of CSs in eyes with ocular toxoplasmosis were evaluated using SD-OCT. Out of 80 eyes included in the study, 17 eyes of 15 patients had cystoid changes seen in the macula. Among these, CME (6/80, or 7.5%), cystoid degeneration (12/80, 15%) HORC (2/80, or 2.5%), and outer retinal tubulation (3/80, or 3.75%) due to AMD were observed.

CME is considered a severe complication of ocular toxoplasmosis [11], [12]. FA has been the traditional gold standard for the detection of CME in the past years; however, with the advent of OCT, more CME could be possibly observed with better reproducibility [8], [13]. Although CME in ocular toxoplasmosis was reported in previous publications, none of them evaluated CME with SD-OCT [14], [15], [16]. Thus, in the current study, we tried to use SD-OCT to maximize the detection of CME. As shown in our study, out of a total of 80 eyes with ocular toxoplasmosis with SD-OCT examinations, 6 eyes were present with CME. Although with the application of SD-OCT, more eyes with CME probably could have been detected, our study had shown that CME is still rather uncommon [14], [15], [16].

Outer retinal tubulation was first reported by Zweifel and colleges and is now a well-accepted concept [5], [17]. Degenerating photoreceptors may become arranged in a circular or ovoid fashion during a process of tubulation in AMD or other atrophic disorders or in eyes with CNV after treatment with anti-angiogenic agents. In our study, all 3 eyes demonstrated outer retinal tubulation were due to late degenerative change after treatment of CNV secondary to AMD in eyes with ocular toxoplasmosis. However, outer retinal tubulation as an independent feature secondary to ocular toxoplasmosis alone without AMD was not observed in our large case series.

HORC was observed during the acute process of active ocular toxoplasmosis in two cases. In the eye with HORC in Patient 1, a membranous structure was seen at the outer aspect in a number of adjacent OCT B-scans. We hypothesize that this structure represents the tissue between ELM and the inner boundary of the RPE (i.e., PR), which further suggests that the lesion represents an intraretinal rather than a subretinal fluid accumulation. A number of other findings support this theory. Firstly, the membranous structure has a uniform thickness (mean 67.1 µm, measured in Figure 2B–E, G), suggesting retinal tissue rather than a chronic inflammatory membrane. Secondly, the thickness of the membranous structure was not significantly different from the PR thickness on either the left (67.0±2.20 µm) or the right (66.1±3.3 µm) side. Thirdly, the lesion thickness was similar to the subfoveal PR thickness in the fellow normal eye (67 µm). In addition, the persistent irregularities in PR and the outer nuclear layer (ONL) seen at follow-up (Figure 2O–Q), even when both the subfoveal morphology and vision had recovered (Figure 2Q), supports our hypothesis that previous abnormalities existed at the level of the outer retina and photoreceptors.

Patient 2 had other unique features: multiple HORC episodes and locations occurred during the disease process. With regard to HORC location at the initial visit, two possibilities were drawn: CS in the outer retina, excluding photoreceptor inner segment (Figure 3fa) and CS in the outer retina, involving photoreceptor inner segment (Figure 3fb). After resolution of the initial HORC, a new HORC appeared at a different location, in the photoreceptor OS. This was further proved by the fact that, in the following visit, at the site where this HORC lesion disappeared, a hyperreflective band was formed in the photoreceptor OS (Figure 3p). The presence of HORC located in the photoreceptor OS of eyes with ocular toxoplasmosis has not been described clinically; however, it has previously been reported in an animal model of ocular toxoplasmosis using light microscopy, in which, enlargement of the interstitial space in photoreceptor OS and cystoid changes in OPL were observed [18]. The findings from Patient 2 in our study that HORC presented in the OS along with CSs in the OPL was consistent with their report; and thus offered clinical evidence for the findings in the experimental animal model. A second unique feature of Case 2 was the obvious PR elongation (Figure 3D–I) and PR splitting (first at the initial HORC detection and subsequently at new HORC appearance in the OS; Figure 3F, 3H, 3M). This splitting of the PR and formation of a membranous structure might happen through binding with inflammatory products like fibrin [19]. Although the exact role of these novel findings in the evolution of these lesions is unknown, our report expanded the clinical spectrum of ocular toxoplasmosis and thus, may aid the further understanding of the pathogenesis of ocular toxoplasmosis.

On OCT in this study, CSs seen in the macula without increased retinal thickness (edema) or outer retinal tubulation were considered cystoid degeneration. This finding has been reported before in eyes with ocular toxoplasmosis, particularly in those with scarred down lesions [11], [20], [21], [22]; however, without detailed OCT analysis. In our case series, 12 eyes with cystoid degeneration seen on SD-OCT, among which, 9 were accompanied by old scars, consistent with previous reports [11], [20], [21], [22]. However, another 3 lesions were present in an otherwise normal macula (within normal limits on both dilated fundoscopy and macular OCT examinations). A common finding from these eyes was that cystoid degeneration presented in the inner retina adjacent to a blood vessel. Normal or almost normal retinal structure has previously been seen in histologic or ophthalmoscopic observations both in human and animal models in eyes infected by T. gondii [23], [24], [25], [26]. Since Toxoplasma parasites can enter into the eye through the vasculature [27], it can produce a pattern of parasite distribution that is restricted to the inner retina [18]. In addition, cystoid changes have also reported adjacent to parasites in an animal model of ocular toxoplasmosis [18]. Thus, our hypothesis is that CSs next to vessels in the inner retina may be due to parasite release from the retinal vessels, or a sign of local inflammation (but without sufficient severity to cause obvious retinal morphological changes). However, if our hypothesis is correct, caution must be paid to possible future reactivation of parasites in these cases, because active lesions remote from old scars could be caused by rupture of a pseudocyst located in apparently normal retina [24]. Thus, more often clinical monitor of disease activation might be needed for these eyes. Further studies are needed to verify the relevance of these SD-OCT features in assisting with the diagnosis and management of ocular toxoplasmosis.

Our study has limitations. Interpretation of PR features in Patients with HORC on OCT is limited by blurred view of the line representing the photoreceptor inner segment-outer segment (IS-OS) junction and ELM. This may reflect poor visibility due to inflammation of the anterior chamber and vitreous opacity in the process of active uveitis, or a limitation of current SD-OCT systems. However, the lines representing the ELM and photoreceptor IS-OS junction in the areas not covered by HORC were of great help in differentiating these boundaries. The other clear limitation is the retrospective study design, but we hoped to include a maximum number of eyes this way, as our goal was to identify morphological phenotypes of ocular toxoplasmosis lesions on SD-OCT. We realize that the process of lesion formation and their response to treatment would best be studied in a prospective series.

## Conclusion

In this study, we demonstrated different phenotypes of CSs in eyes with ocular toxoplasmosis. SD-OCT is not only able to detect common CSs next to the old scars, but also able to detect rare occurrences, e.g. HORC. Identification of rare morphological cystoid features (HORC with/without photoreceptor enlongation or splitting) on clinical examination had provided evidence to previous experimental models, which may also expand the clinical spectrum of the disease. Cystoid degeneration in the inner retina next to the retinal vessels in otherwise “normal” looking macula was observed, which may suggest more often clinical evaluation for those patients. Further studies are needed to verify the relevance of cystoid features seen on SD-OCT in assisting with the diagnosis and management of ocular toxoplasmosis.
